# Rapid Genotyping of Swine Influenza Viruses

**DOI:** 10.3201/eid1704.101726

**Published:** 2011-04

**Authors:** Polly W.Y. Mak, Chloe K.S. Wong, Olive T.W. Li, Kwok Hung Chan, Chung Lam Cheung, Edward S. Ma, Richard J. Webby, Yi Guan, Joseph S. Malik Peiris, Leo L.M. Poon

**Affiliations:** Author affiliations: The University of Hong Kong, Hong Kong Special Administrative Region, People’s Republic of China (P.W.Y. Mak, C.K.S. Wong, O.T.W. Li, K.H. Chan, C.L. Cheung, E.S. Ma, Y. Guan, J.S.M. Peiris, L.L.M. Poon);; St. Jude Children’s Research Hospital, Memphis, Tennessee, USA (R.J. Webby);; Hong Kong University–Pasteur Research Centre, Hong Kong (J.S.M. Peiris)

**Keywords:** Swine influenza, reassortment, genotyping, influenza, viruses, dispatch

## Abstract

The emergence of pandemic (H1N1) 2009 virus highlighted the need for enhanced surveillance of swine influenza viruses. We used real-time reverse–transcription PCR–based genotyping and found that this rapid and simple genotyping method may identify reassortants derived from viruses of Eurasian avian-like, triple reassortant-like, and pandemic (H1N1) 2009 virus lineages.

Co-infection of influenza A viruses enables viral gene reassortments, thereby generating progeny viruses with novel genotypes. Such reassortants may pose a serious public health threat, as exemplified by the emergence of pandemic influenza (H1N1) in 2009 (*1*). Transmission of pandemic (H1N1) 2009 virus from humans to pigs has been reported (*2*–*5*). We recently identified a reassortment between pandemic (H1N1) 2009 virus and swine influenza viruses in pigs (*6*). These results emphasize the potential role of pigs as a mixing vessel for influenza viruses and the need for screening tests that can identify major reassortment events in pigs.

We previously developed 8 monoplex SYBR green–based quantitative reverse transcription–PCRs to detect all 8 gene segments derived from the pandemic (H1N1) 2009 virus or virus segments that are closely related to this lineage (i.e., neuraminidase [NA] and matrix protein from the Eurasian avian-like swine linage and polymerase basic protein [PB] 2, PB1, polymerase acidic protein [PA], hemagglutinin [HA], nucleocapsid protein [NP], and nonstructural protein [NS]) from triple reassortant swine linage (*5*). Using these PCRs, we identified swine viruses of atypical genotypes. However, with the exception of the HA-specific assay, the melting-curve signals of pandemic (H1N1) 2009 virus may be indistinguishable from the positive signals generated from its sister clade as indicated above. To differentiate between these closely related groups of viruses, we further optimized these assays by adding sequence-specific hydrolysis probes in the SYBR green assays.

## The Study

For this study, all SYBR green assays were modified from the previously described assays (*5*), with the exception of the reverse primers for the newly designed PB1 and NS segments (Table 1). The subtype H1N1 swine influenza viruses isolated in Hong Kong during the past few years were mainly derived from the Eurasian avian-like swine lineage (*6*,*7*). To generate more precise genotyping data for our ongoing surveillance, the NA segment–specific assay was specifically designed to react with the pandemic (H1N1) 2009 virus and a portion of Eurasian avian-like swine viruses that are circulating in southeastern China ( Technical Appendix Figure 1). To avoid overlapping the emission spectrum of SYBR green, we labeled all pandemic (H1N1) 2009 virus–specific hydrolysis probes (Integrated DNA Technologies, Inc., Coralville, IA, USA) with cyanine 5 (Cy5) and Black Hole Quencher-2 dyes at their 5′ and 3′ ends, respectively (Table 1). To enable use of short oligonucleotide sequences without compromising the annealing temperature of these probes, we modified the probes with locked nucleic acids (*7*). RNA extraction and complimentary DNA synthesis were identical to the protocols described (*5*,*8*). One microliter of 10-fold diluted complimentary DNA sample was amplified in a 20-µL reaction containing 10 µL of Fast SYBR Green Master Mix (Applied Biosystems, Foster City, CA, USA) and the corresponding primer probe set (0.5 µmol/L each). All reactions were optimized and performed simultaneously in a 7500 Sequence Detection System (Applied Biosystems) with the following conditions: 20 s at 95°C, followed by 30 cycles of 95°C for 3 s and 62°C for 30 s. SYBR green and Cy5 signals from the same reaction were captured simultaneously at the end of each amplification cycle. The expected PCR results of virus segment derived from different swine viral lineages are shown in Table 2.

**Table 1:** Primer–probe sets selective for pandemic (H1N1) 2009 virus gene segments*

Segment	Primer and probe†	Sequence, 5′ → 3′‡
PB2	PB2-1877F§	AACTTCTCCCCTTTGCTGCT
	PB2-2062R§	GATCTTCAGTCAATGCACCTG
	PB2-2028RP	Cy5-AACTGTAAGTCGTTTGGT-BHQ2
PB1	PB1-825F§	ACAGTCTGGGCTCCCAGTA
	PB1-1023R	GAACCACTCGGGTTGATTTCTG
	PB1-863FP	Cy5-CCAAACTGGCAAATG-BHQ2
PA	PA-821F§	GCCCCCTCAGATTGCCTG
	PA-1239R§	GCTTGCTAGAGATCTGGGC
	PA-844FP	Cy5-CCTCTTTGCCATCAGC-BHQ2
HA	HA-398F§	GAGCTCAGTGTCATCATTTGAA
	HA-570R§	TGCTGAGCTTTGGGTATGAA
	HA-470FP	Cy5-CAAAGGTGTAACGGCA-BHQ2
NP	NP-593F§	TGAAAGGAGTTGGAACAATAGCAA
	NP-942R§	GACCAGTGAGTACCCTTCCC
	NP-872RP	Cy5-AGGCAGGATTTATGTG-BHQ2
NA	NA-163F§	CATGCAATCAAAGCGTCATT
	NA-268R§	ACGGAAACCACTGACTGTCC
	NA-248RP	Cy5-AGCAGCAAAGTTGGTG-BHQ2
M	M-504F§	GGTCTCACAGACAGATGGCT
	M-818R§	GATCCCAATGATATTTGCTGCAATG
	M-530FP	Cy5-ACCAATCCACTAATCAGG-BHQ2
NS	NS-252F§	ACACTTAGAATGACAATTGCATCTGT
	NS-345R	GCATGAGCATGAACCAGTCTCG
	NS-288FP	Cy5-CGCTACCTTTCTGACAT/BHQ2/

*PB, polymerase basic protein; PA, polymerase acidic protein; HA, hemagglutinin; NP, nucleocapsid protein; NA, neuraminidase; M, matrix protein; NS, nonstructural protein; Cy5, cyanine 5; BHQ2, Black Hole Quencher 2. †Number represents nucleotide position of the first base in the target sequence (cRNA sense). ‡Locked nucleic acid–modified bases are underlined. §Primers adapted from the assays as previously described (5,6).

**Table 2:** Summary of expected genotyping results of swine and human influenza viruses*

Virus	PB2	PB1	PA	HA	NP	NA†	M	NS
Pandemic (H1N1) 2009	**+ +**	**+ +**	**+ +**	**+ +**	**+ +**	**+ +**	**+ +**	**+ +**
Swine Eurasian avian-like	**– –**	**– –**	**– –**	**– –**	**– –**	**– +**	**– +**	**– –**
Swine triple reassortant	**– +**	**– +**	**– +**	**– +**	**– +**	**– –**	**– –**	**– +**
Human seasonal subtypes H1 and H3	**– –**	**– –**	**– –**	**– –**	**– –**	**– –**	**– –**	**– –**

*PB, polymerase basic protein; PA, polymerase acidic protein; HA, hemagglutinin; NP, nucleocapsid protein; NA, neuraminidase; M, matrix protein; NS, nonstructural protein. Red symbols indicate pandemic (H1N1) 2009 virus–specific probe results; black symbols indicate SYBR Green results; gray shading indicates sister clade of pandemic (H1N1) 2009 virus for each virus segment. †N2 and some of the N1 within swine Euarasian avian-like lineage are expected to be double negative in the NA test.

The dissociation kinetics of PCR amplicons were studied by a melting curve analysis at the end of the PCR (60°C–95°C; temperature increment 0.1°C/s). We also tested various probe and SYBR green concentrations under different PCR conditions. The condition described above gave the most robust and consistent DNA amplification (data not shown). We tested 31 human pandemic (H1N1) 2009 and 63 human seasonal influenza viruses (33 subtype H1N1, 30 subtype H3N2) as controls. As expected, all human pandemic influenza viruses were double positive (i.e., positive with SYBR green and Cy5) and all seasonal influenza samples were double negative in all 8 assays.

To evaluate the sensitivity of the assays, we tested serial diluted plasmid DNA of the corresponding segments of influenza A/California/4/2009 virus as a standard. The fluorescent signals generated from the SYBR green reporter dye in all assays were highly similar to those previously reported (*5*), and the modified assays had a linear dynamic detection range from 10^2^ to 10^8^ copies/reaction (Technical Appendix Figure 2). As expected, the threshold cycle values deduced from the Cy5 reporter signal were generally higher than those from the SYBR green reporter (Technical Appendix Figure 2) (*9*). This finding can be partly explained by the nature of these 2 kinds of real-time PCR chemistries: a single Cy5 fluorophore of the hydrolysis probe was released from quenching for each amplicon synthesized while multiple SYBR green dyes bound to a single amplicon (*10*). After 35 PCR amplification cycles, the linear dynamic detection range of Cy5 signals generated from these reactions was 10^2^ to 10^8^ copies/reaction (data not shown). However, to avoid nonspecific SYBR green signals, we purposely limited the number of amplification cycles to 30.

Using these assays, we tested 41 swine virus isolates collected during January 2009–January 2010. In all 8 reactions, 10 pandemic (H1N1) 2009 virus samples transmitted from humans to pigs (*6*) were double positive (Technical Appendix Figure 1, pink). In these assays, gene segments of another 31 swine isolates were either SYBR green positive/Cy5 negative (Technical Appendix Figure 1, yellow) or double negative (Technical Appendix Figure 1, green), indicating that these virus segments were derived from the sister clade of pandemic (H1N1) 2009 virus or other swine lineages (except NA), respectively. For example, the reassortant of pandemic (H1N1) 2009 virus (A/swine/Hong Kong/201/2010 [H1N1]) was double positive for NA, double negative for HA and matrix protein, and SYBR green positive/Cy5 negative for PB2, PB1, PA, NP, and NS (Figure; other data not shown). All genotyping results of the studied viruses were consistent with results of previous phylogenetic analyses (*5*,*6*), indicating that our modified probes and SYBR green assays can provide more accurate genotyping results. With these genotyping data, viruses with atypical positive signal patterns might suggest a novel viral reassortment event and can be highlighted for investigation with sequencing-based methods.

**Figure:**
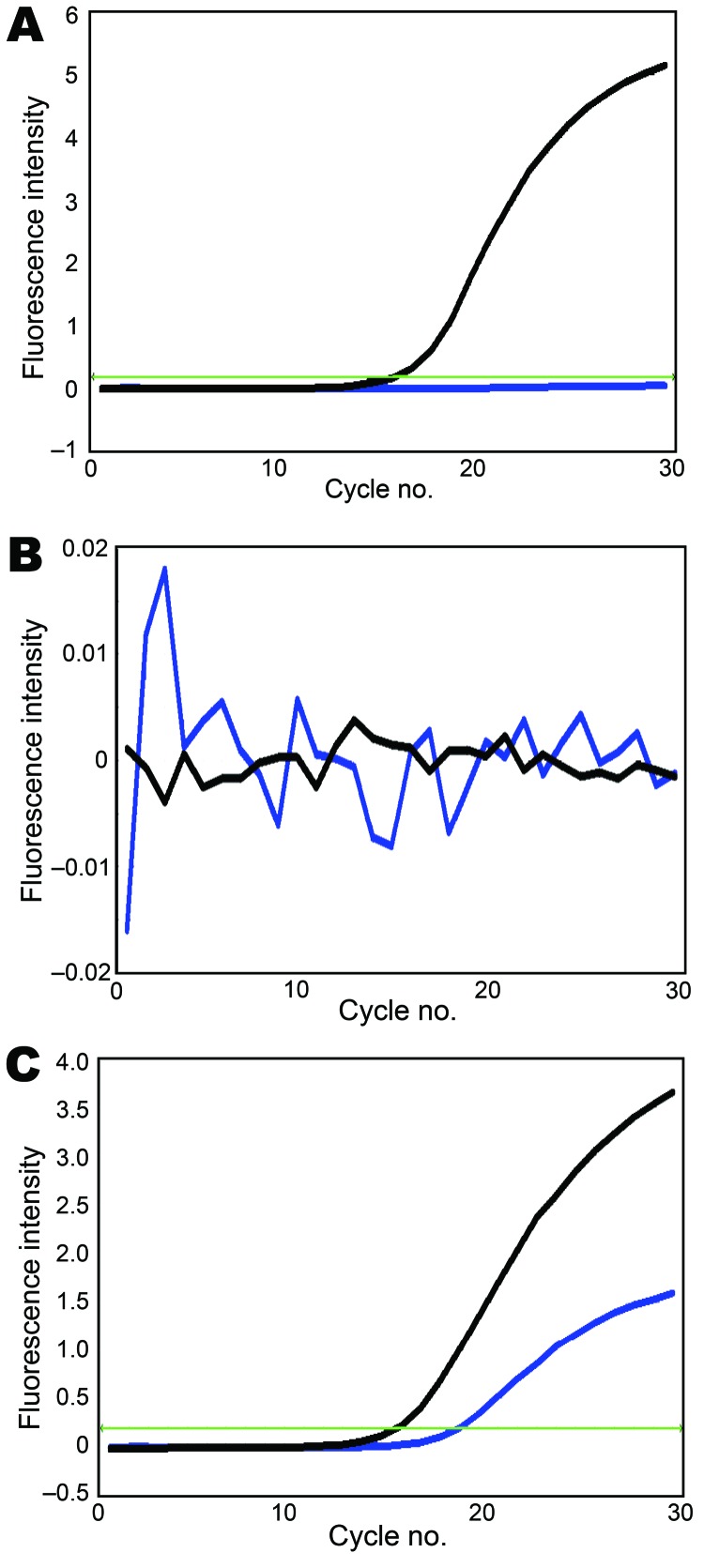
Genotyping of A) polymerase acidic protein, B) hemagglutinin, and C) neuraminidase segments of A/swine/Hong Kong/201/2010 influenza (H1N1) virus. Black line, amplification signal of SYBR green dye; blue line, amplification signal of cyanine 5 dye; green line, threshold level. The x-axis denotes the cycle number of a quantitative PCR assay, and the y-axis denotes the fluorescence intensity over the background.

To demonstrate the potential use of these assays in studying swine viruses circulating in other geographic locations, we tested 7 recent swine isolates (1 pandemic influenza subtype H1N1, 4 subtype H1N2, and 2 subtype H3N2) collected in the United States. Genotyping results agreed 100% with data deduced from sequence analyses (Technical Appendix Table 1). We also analyzed all 436 contemporary (2008–2010) US swine virus segments available from the National Center for Biotechnology Information influenza virus sequence database. On the basis of the in silico analysis of sequences targeted by our primers and probes, 95% of the sequences (n = 413) are predicted to yield the expected results (Technical Appendix Table 2).

## Conclusions

The emergence of pandemic (H1N1) 2009 has highlighted the need for global systematic influenza surveillance in swine. Our results demonstrated that the addition of locked nucleic acid hydrolysis probes specific for pandemic (H1N1) 2009 virus into previously established SYBR green assays can help differentiate segments of pandemic (H1N1) 2009, Eurasian avian-like, and triple reassortant virus lineages. These assays might provide a rapid and simple genotyping method for identifying viruses that need to be fully genetically sequenced and characterized. They may also help provide better understanding of the viral reassortment events and viral dynamics in pigs. Although at present, genes derived from human seasonal viruses cannot be characterized with our modified assays, the performance of our assays warrants similar investigations for genotyping human influenza viruses.

## Supplementary Material

Technical Appendix Genotyping results , viral sequences, and Combined SYBR green/hydrolysis probe quantitative RT-PCR assays for swine influenza virus
