# Supplementary material for: Rapid Genotyping of Swine Influenza Viruses
Source: Emerg Infect Dis. 2011 Apr;17(4):691–4. doi: 10.3201/eid1704.101726 (PMC3377423; doi:10.3201/eid1704.101726)
Supplement: Technical Appendix — Genotyping results , viral sequences, and Combined SYBR green/hydrolysis probe quantitative RT-PCR assays for swine influenza virus [file 10-1726-Techapp.pdf]

Article DOI: 10.3201/eid1704.101726

Rapid Genotyping of Swine Influenza Viruses

Technical Appendix

Technical Appendix Table 1. Genotyping results of contemporary (2007–2008) US swine influenza viruses.

Virus[1]	Subtype	PB2		PB1		PA		HA		NP		NA		M		NS	
		L	R[2]	L	R[2]	L	R[2]	L	R[2]	L	R[2]	L	R[2]	L	R[2]	L	R[2]
A/Sw/Oklahoma/ 008722/2007 (670136)	H3N2	TR	- +	TR	- +	TR	- +	H3	- -	TR	- +	N2	- -	TR	- -	TR	- +
A/Sw/Oklahoma/ 011506/2007 (670139)	H3N2	TR	- +	TR	- +	TR	- +	H3	- -	TR	- +	N2	- -	TR	- -	TR	- +
A/Sw/Texas/ 008648/2008 (670134)	H1N2	TR	- +	TR	- +	TR	- +	Human H1	- -	TR	- +	N2	- -	TR	- -	TR	- +
A/Sw/Oklahoma/ 032726/2008 (670116)	H1N2	TR	- +	TR	- +	TR	- +	Human H1	- -	TR	- +	N2	- -	TR	- -	TR	- +
A/Sw/Texas/ 050593/2008 (670115)	H1N2	TR	- +	TR	- +	TR	- +	Human H1	- -	TR	- +	N2	- -	TR	- -	TR	- +
A/Sw/Texas/ 050625/2008 (670132)	H1N2	TR	- +	TR	- +	TR	- +	Human H1	- -	TR	- +	N2	- -	TR	- -	TR	- +
A/Sw/Iowa/ 14-1010/2009	H1N1	pH1N1	+ +	pH1N1	+ +	pH1N1	+ +	pH1N1	+ +	pH1N1	+ +	pH1N1	+ +	pH1N1	+ +	pH1N1	+ +

*L, lineage; R, results.

[1] All viruses were isolated from pigs in US. Except the pandemic H1N1 (A/Sw/Iowa/14-1010/2009; Webby RJ, unpublished data), the identity of each virus in the NCBI Taxonomy ID database is shown.

[2]Blue signs represent pandemic H1N1-specific probe results while black signs represent SYBR Green results.

Technical Appendix Table 2. Contemporary US swine viral sequences (2008-2010) downloaded from Influenza Virus Sequence Database (N=436)*

**PB2 (N=40)**
Influenza A virus (A/swine/Illinois/Sg-00443/2008(H1N1))
Influenza A virus (A/swine/Illinois/Sg-00444/2008(H1N1))
Influenza A virus (A/swine/Illinois/Sg-00445/2008(H1N1))
Influenza A virus (A/swine/Iowa/02039/2008(H1N2))
Influenza A virus (A/swine/Iowa/02096/2008(H1N1))
Influenza A virus (A/swine/Kansas/015252/2009(H3N2))
Influenza A virus (A/swine/Kentucky/02086/2008(H1N1))
Influenza A virus (A/swine/Minnesota/02011/2008(H1N2))
Influenza A virus (A/swine/Minnesota/02053/2008(H1N1))
Influenza A virus (A/swine/Minnesota/02093/2008(H1N1))
Influenza A virus (A/swine/Missouri/02060/2008(H1N1))
Influenza A virus (A/swine/MN/23506/2009(H1N1))
Influenza A virus (A/swine/Nebraska/02013/2008(H1N1))
Influenza A virus (A/swine/North Carolina/02023/2008(H1N1))
Influenza A virus (A/swine/North Carolina/02084/2008(H1N1))
Influenza A virus (A/swine/North Carolina/R08-001877-D08-013371/2008 (H3N2))
Influenza A virus (A/swine/Ohio/02026/2008(H1N1))
Influenza A virus (A/swine/Oklahoma/001142/2009(H3N2))
Influenza A virus (A/swine/Oklahoma/010226-16/2008(H1N2))
Influenza A virus (A/swine/Oklahoma/010226-17/2008(H1N2))
Influenza A virus (A/swine/Oklahoma/010710-8/2008(H1N2))
Influenza A virus (A/swine/Oklahoma/010710-9/2008(H1N2))
Influenza A virus (A/swine/Oklahoma/011289-10/2008(H1N2))
Influenza A virus (A/swine/Oklahoma/011289-8/2008(H1N2))
Influenza A virus (A/swine/Oklahoma/011289-9/2008(H1N2))
Influenza A virus (A/swine/Oklahoma/011521-4/2008(H1N2))
Influenza A virus (A/swine/Oklahoma/011521-5/2008(H1N2))
Influenza A virus (A/swine/Oklahoma/016179-8/2008(H1N2))
Influenza A virus (A/swine/Oklahoma/016179-9/2008(H1N2))
Influenza A virus (A/swine/Oklahoma/020734-2/2008(H1N2))
Influenza A virus (A/swine/Oklahoma/020734-3/2008(H1N2))
Influenza A virus (A/swine/Oklahoma/020736-1/2008(H1N2))
Influenza A virus (A/swine/Oklahoma/020736-2/2008(H1N2))
Influenza A virus (A/swine/Oklahoma/032726/2008(H1N2))
Influenza A virus (A/swine/Oklahoma/042169/2008(H1N2))
Influenza A virus (A/swine/Oklahoma/053259/2008(H1N2))
Influenza A virus (A/swine/Texas/008648/2008(H1N2))
Influenza A virus (A/swine/Texas/01976/2008(H1N2))
Influenza A virus (A/swine/Texas/050593/2008(H1N2))
Influenza_A_virus_(A/swine/Texas/050625/2008(H1N2))
**PA (N=38)**
Influenza A virus (A/swine/Illinois/Sg-00443/2008(H1N1))
Influenza A virus (A/swine/Illinois/Sg-00444/2008(H1N1))
Influenza A virus (A/swine/Illinois/Sg-00445/2008(H1N1))
Influenza A virus (A/swine/Iowa/02039/2008(H1N2))
Influenza A virus (A/swine/Iowa/02096/2008(H1N1))
**Influenza A virus (A/swine/Kansas/015252/2009(H3N2))**
Influenza A virus (A/swine/Kentucky/02086/2008(H1N1))
**Influenza A virus (A/swine/Minnesota/02053/2008(H1N1))**
Influenza A virus (A/swine/Missouri/02060/2008(H1N1))
Influenza A virus (A/swine/MN/23506/2009(H1N1))
Influenza A virus (A/swine/Nebraska/02013/2008(H1N1))
Influenza A virus (A/swine/North Carolina/02023/2008(H1N1))
Influenza A virus (A/swine/North Carolina/02084/2008(H1N1))

Influenza A virus (A/swine/North Carolina/R08-001877-D08-013371/2008 (H3N2))
Influenza A virus (A/swine/Ohio/02026/2008(H1N1))
Influenza A virus (A/swine/Oklahoma/001142/2009(H3N2))
Influenza A virus (A/swine/Oklahoma/010226-16/2008(H1N2))
Influenza A virus (A/swine/Oklahoma/010226-17/2008(H1N2))
Influenza A virus (A/swine/Oklahoma/010710-8/2008(H1N2))
Influenza A virus (A/swine/Oklahoma/010710-9/2008(H1N2))
Influenza A virus (A/swine/Oklahoma/011289-10/2008(H1N2))
Influenza A virus (A/swine/Oklahoma/011289-8/2008(H1N2))
Influenza A virus (A/swine/Oklahoma/011289-9/2008(H1N2))
Influenza A virus (A/swine/Oklahoma/011521-4/2008(H1N2))
Influenza A virus (A/swine/Oklahoma/011521-5/2008(H1N2))
Influenza A virus (A/swine/Oklahoma/016179-8/2008(H1N2))
Influenza A virus (A/swine/Oklahoma/016179-9/2008(H1N2))
Influenza A virus (A/swine/Oklahoma/020734-2/2008(H1N2))
Influenza A virus (A/swine/Oklahoma/020734-3/2008(H1N2))
Influenza A virus (A/swine/Oklahoma/020736-1/2008(H1N2))
Influenza A virus (A/swine/Oklahoma/020736-2/2008(H1N2))
Influenza A virus (A/swine/Oklahoma/032726/2008(H1N2))
Influenza A virus (A/swine/Oklahoma/042169/2008(H1N2))
Influenza A virus (A/swine/Oklahoma/053259/2008(H1N2))
Influenza A virus (A/swine/Texas/008648/2008(H1N2))
Influenza A virus (A/swine/Texas/01976/2008(H1N2))
Influenza A virus (A/swine/Texas/050593/2008(H1N2))
Influenza A virus (A/swine/Texas/050625/2008(H1N2))

PB1 (N=35)

Influenza A virus (A/swine/Illinois/Sg-00443/2008(H1N1))
Influenza A virus (A/swine/Iowa/02039/2008(H1N2))
Influenza A virus (A/swine/Kansas/015252/2009(H3N2))
Influenza A virus (A/swine/Kentucky/02086/2008(H1N1))
Influenza A virus (A/swine/Minnesota/02011/2008(H1N2))
Influenza A virus (A/swine/Minnesota/02053/2008(H1N1))
Influenza A virus (A/swine/Minnesota/02093/2008(H1N1))
Influenza A virus (A/swine/Missouri/02060/2008(H1N1))
Influenza A virus (A/swine/MN/23506/2009(H1N1))
Influenza A virus (A/swine/Nebraska/02013/2008(H1N1))
Influenza A virus (A/swine/North Carolina/02023/2008(H1N1))
Influenza A virus (A/swine/North Carolina/02084/2008(H1N1))
Influenza A virus (A/swine/North Carolina/R08-001877-D08-013371/2008 (H3N2))
Influenza A virus (A/swine/Ohio/02026/2008(H1N1))
Influenza A virus (A/swine/Oklahoma/001142/2009(H3N2))
Influenza A virus (A/swine/Oklahoma/010226-16/2008(H1N2))
Influenza A virus (A/swine/Oklahoma/010226-17/2008(H1N2))
Influenza A virus (A/swine/Oklahoma/010710-8/2008(H1N2))
Influenza A virus (A/swine/Oklahoma/010710-9/2008(H1N2))
Influenza A virus (A/swine/Oklahoma/011289-10/2008(H1N2))
Influenza A virus (A/swine/Oklahoma/011289-8/2008(H1N2))
Influenza A virus (A/swine/Oklahoma/011289-9/2008(H1N2))
Influenza A virus (A/swine/Oklahoma/011521-4/2008(H1N2))
Influenza A virus (A/swine/Oklahoma/011521-5/2008(H1N2))
Influenza A virus (A/swine/Oklahoma/016179-8/2008(H1N2))
Influenza A virus (A/swine/Oklahoma/016179-9/2008(H1N2))
Influenza A virus (A/swine/Oklahoma/020734-2/2008(H1N2))
Influenza A virus (A/swine/Oklahoma/020734-3/2008(H1N2))
Influenza A virus (A/swine/Oklahoma/020736-1/2008(H1N2))
Influenza A virus (A/swine/Oklahoma/020736-2/2008(H1N2))
Influenza A virus (A/swine/Oklahoma/032726/2008(H1N2))

Influenza A virus (A/swine/Oklahoma/042169/2008(H1N2))
Influenza A virus (A/swine/Oklahoma/053259/2008(H1N2))
Influenza A virus (A/swine/Texas/008648/2008(H1N2))
Influenza A virus (A/swine/Texas/050625/2008(H1N2))
**NP (N=35)**
Influenza A virus (A/swine/Iowa/02039/2008(H1N2))
Influenza A virus (A/swine/Kansas/015252/2009(H3N2))
Influenza A virus (A/swine/Kentucky/02086/2008(H1N1))
Influenza A virus (A/swine/Minnesota/02011/2008(H1N2))
Influenza A virus (A/swine/Minnesota/02053/2008(H1N1))
Influenza A virus (A/swine/Minnesota/02093/2008(H1N1))
Influenza A virus (A/swine/Missouri/02060/2008(H1N1))
Influenza A virus (A/swine/MN/23506/2009(H1N1))
Influenza A virus (A/swine/Nebraska/02013/2008(H1N1))
Influenza A virus (A/swine/North Carolina/02023/2008(H1N1))
Influenza A virus (A/swine/North Carolina/R08-001877-D08-013371/2008 (H3N2))
Influenza A virus (A/swine/Ohio/02026/2008(H1N1))
Influenza A virus (A/swine/Oklahoma/001142/2009(H3N2))
Influenza A virus (A/swine/Oklahoma/010226-16/2008(H1N2))
Influenza A virus (A/swine/Oklahoma/010226-17/2008(H1N2))
Influenza A virus (A/swine/Oklahoma/010710-8/2008(H1N2))
Influenza A virus (A/swine/Oklahoma/010710-9/2008(H1N2))
Influenza A virus (A/swine/Oklahoma/011289-10/2008(H1N2))
Influenza A virus (A/swine/Oklahoma/011289-8/2008(H1N2))
Influenza A virus (A/swine/Oklahoma/011289-9/2008(H1N2))
Influenza A virus (A/swine/Oklahoma/011521-4/2008(H1N2))
Influenza A virus (A/swine/Oklahoma/011521-5/2008(H1N2))
Influenza A virus (A/swine/Oklahoma/016179-8/2008(H1N2))
Influenza A virus (A/swine/Oklahoma/016179-9/2008(H1N2))
Influenza A virus (A/swine/Oklahoma/020734-2/2008(H1N2))
Influenza A virus (A/swine/Oklahoma/020734-3/2008(H1N2))
Influenza A virus (A/swine/Oklahoma/020736-1/2008(H1N2))
Influenza A virus (A/swine/Oklahoma/020736-2/2008(H1N2))
Influenza A virus (A/swine/Oklahoma/032726/2008(H1N2))
Influenza A virus (A/swine/Oklahoma/042169/2008(H1N2))
Influenza A virus (A/swine/Oklahoma/053259/2008(H1N2))
Influenza A virus (A/swine/Texas/008648/2008(H1N2))
Influenza A virus (A/swine/Texas/01976/2008(H1N2))
Influenza A virus (A/swine/Texas/050593/2008(H1N2))
Influenza A virus (A/swine/Texas/050625/2008(H1N2))
**HA (N=87)**
Influenza A virus (A/swine/IL/10-001550/2009(H1N1))
Influenza A virus (A/swine/IL/3910/2010(H1N1))
Influenza A virus (A/swine/Iowa/03032/2010(H1N1))
Influenza A virus (A/swine/IL/12660/2010(H1N1))
Influenza A virus (A/swine/Kentucky/02086/2008(H1N1))
Influenza A virus (A/swine/MN/8762-1/2010(H1N1))
Influenza A virus (A/swine/Illinois/03037/2010(H1N1))
Influenza A virus (A/swine/OR/10-004060/2009(H1N1))
Influenza A virus (A/swine/Minnesota/02976/2010(H1N1))
Influenza A virus (A/swine/IL/27486-1/2010(H1N1))
Influenza A virus (A/swine/Iowa/02096/2008(H1N1))
Influenza A virus (A/swine/IL/25399-3/2010(H1N1))
Influenza A virus (A/swine/Iowa/02999/2010(H1N1))
Influenza A virus (A/swine/IL/35573/2009(H1N1))
Influenza A virus (A/swine/Iowa/03031/2010(H1N1))

Influenza A virus (A/swine/North Carolina/02023/2008(H1N1))
Influenza A virus (A/swine/MN/23506/2009(H1N1))
Influenza A virus (A/swine/MN/8761/2010(H1N1))
Influenza A virus(A/swine/Iowa/46519-2/2008(H1N1))
Influenza A virus (A/swine/MN/8762-2/2010(H1N1))
Influenza A virus (A/swine/SD/1/2010(H1N1))
Influenza A virus(A/swine/Iowa/46519-4/2008(H1N1))
Influenza A virus (A/swine/MO/23881/2010(H1N1))
Influenza A virus(A/swine/Iowa/46519-3/2008(H1N1))
Influenza A virus (A/swine/NC/19646/2010(H1N1))
Influenza A virus (A/swine/Minnesota/03025/2010(H1N1))
Influenza A virus (A/swine/NC/34752/2009(H1N1))
Influenza A virus (A/swine/Missouri/02060/2008(H1N1))
Influenza A virus (A/swine/IL/32974/2009(H1N1))
Influenza A virus (A/swine/IL/5265-2/2010(H1N1))
Influenza A virus (A/swine/Ohio/02026/2008(H1N1))
Influenza A virus (A/swine/IL/17315-3/2010(H1N1))
Influenza A virus (A/swine/IL/5265-1/2010(H1N1))
Influenza A virus (A/swine/IN/17311/2010(H1N1))
Influenza A virus (A/swine/IL/25399-4/2010(H1N1))
Influenza A virus (A/swine/IL/17315-1/2010(H1N1))
Influenza A virus (A/swine/IL/10-001551-2/2009(H1N1))
Influenza A virus (A/swine/IL/25399-2/2010(H1N1))
Influenza A virus(A/swine/Missouri/46519-5/2009(H1N1))
Influenza A virus (A/swine/Indiana/27007/2009(H1N1))
Influenza A virus (A/swine/Nebraska/02013/2008(H1N1))
Influenza A virus (A/swine/NC/13598/2010(H1N1))
Influenza A virus (A/swine/MO/15534/2010(H1N1))
Influenza A virus (A/swine/IL/35572/2009(H1N1))
Influenza A virus (A/swine/MO/17314/2010(H1N1))
Influenza A virus (A/swine/IL/27486-2/2010(H1N1))
Influenza A virus (A/swine/IL/10-001551-1/2009(H1N1))
Influenza A virus (A/swine/NC/34543/2009(H1N1))
Influenza A virus (A/swine/Iowa/02039/2008(H1N2))
Influenza A virus (A/swine/Iowa/02998/2010(H1N2))
Influenza A virus (A/swine/Minnesota/02093/2008(H1N1))
Influenza A virus (A/swine/Minnesota/03043/2010(H1N2))
Influenza A virus (A/swine/Minnesota/02011/2008(H1N2))
Influenza A virus (A/swine/Minnesota/02053/2008(H1N1))
Influenza A virus (A/swine/Minnesota/03023/2010(H1N2))
Influenza A virus (A/swine/Oklahoma/016179-9/2008(H1N2))
Influenza A virus (A/swine/Minnesota/03000/2010(H1N1))
Influenza A virus (A/swine/Oklahoma/020734-3/2008(H1N2))
Influenza A virus (A/swine/Oklahoma/016179-8/2008(H1N2))
Influenza A virus (A/swine/Oklahoma/011289-8/2008(H1N2))
Influenza A virus (A/swine/Oklahoma/011521-4/2008(H1N2))
Influenza A virus (A/swine/Oklahoma/010710-9/2008(H1N2))
Influenza A virus (A/swine/Oklahoma/010226-16/2008(H1N2))
Influenza A virus (A/swine/Texas/01976/2008(H1N2))
Influenza A virus (A/swine/Oklahoma/010710-8/2008(H1N2))
Influenza A virus (A/swine/SD/31813/2009(H1N2))
Influenza A virus (A/swine/Oklahoma/011289-10/2008(H1N2))
Influenza A virus (A/swine/Oklahoma/020736-1/2008(H1N2))
Influenza A virus (A/swine/Oklahoma/010226-17/2008(H1N2))
Influenza A virus (A/swine/Oklahoma/020734-2/2008(H1N2))
Influenza A virus (A/swine/Illinois/03036/2010(H1N2))
Influenza A virus (A/swine/Illinois/03033/2010(H1N2))

Influenza A virus (A/swine/Iowa/03026/2010(H1N2))
Influenza A virus (A/swine/Oklahoma/032726/2008(H1N2))
Influenza A virus (A/swine/Oklahoma/042169/2008(H1N2))
Influenza A virus (A/swine/Oklahoma/053259/2008(H1N2))
Influenza A virus (A/swine/Texas/050593/2008(H1N2))
Influenza A virus (A/swine/Oklahoma/011289-9/2008(H1N2))
Influenza A virus (A/swine/Missouri/03035/2010(H1N2))
Influenza A virus (A/swine/Oklahoma/011521-5/2008(H1N2))
Influenza A virus (A/swine/Texas/008648/2008(H1N2))
Influenza A virus (A/swine/Oklahoma/020736-2/2008(H1N2))
Influenza A virus (A/swine/Texas/050625/2008(H1N2))
Influenza A virus (A/swine/North Carolina/R08-001877-D08-013371/2008 (H3N2))
Influenza A virus (A/swine/Kansas/015252/2009(H3N2))
Influenza A virus (A/swine/Minnesota/03008/2010(H3N2))
Influenza A virus (A/swine/Oklahoma/001142/2009(H3N2))
**NA (N=73)**
Influenza A virus (A/swine/IL/10-001550/2009(H1N1))
Influenza A virus (A/swine/IL/10-001551-1/2009(H1N1))
Influenza A virus (A/swine/IL/10-001551-2/2009(H1N1))
**Influenza A virus (A/swine/IL/12660/2010(H1N1))**
Influenza A virus (A/swine/IL/17315-1/2010(H1N1))
Influenza A virus (A/swine/IL/17315-3/2010(H1N1))
Influenza A virus (A/swine/IL/25399-2/2010(H1N1))
Influenza A virus (A/swine/IL/25399-3/2010(H1N1))
Influenza A virus (A/swine/IL/25399-4/2010(H1N1))
Influenza A virus (A/swine/IL/27486-1/2010(H1N1))
Influenza A virus (A/swine/IL/27486-2/2010(H1N1))
Influenza A virus (A/swine/IL/32974/2009(H1N1))
Influenza A virus (A/swine/IL/35572/2009(H1N1))
Influenza A virus (A/swine/IL/35573/2009(H1N1))
**Influenza A virus (A/swine/IL/3910/2010(H1N1))**
Influenza A virus (A/swine/IL/5265-1/2010(H1N1))
Influenza A virus (A/swine/IL/5265-2/2010(H1N1))
Influenza A virus (A/swine/IN/17311/2010(H1N1))
Influenza A virus (A/swine/Indiana/27007/2009(H1N1))
Influenza A virus (A/swine/MO/17314/2010(H1N1))
Influenza A virus (A/swine/MO/23881/2010(H1N1))
Influenza A virus (A/swine/NC/13598/2010(H1N1))
Influenza A virus (A/swine/NC/19646/2010(H1N1))
**Influenza A virus (A/swine/MN/8761/2010(H1N1))**
**Influenza A virus (A/swine/MN/8762-1/2010(H1N1))**
**Influenza A virus (A/swine/MN/8762-2/2010(H1N1))**
Influenza A virus (A/swine/MO/15534/2010(H1N1))
Influenza A virus (A/swine/NC/34543/2009(H1N1))
Influenza A virus (A/swine/NC/34752/2009(H1N1))
Influenza A virus (A/swine/Minnesota/02093/2008(H1N1))
Influenza A virus (A/swine/Kentucky/02086/2008(H1N1))
Influenza A virus(A/swine/Iowa/46519-3/2008(H1N1))
Influenza A virus (A/swine/OR/10-004060/2009(H1N1))
Influenza A virus (A/swine/SD/1/2010(H1N1))
Influenza A virus (A/swine/Missouri/02060/2008(H1N1))
Influenza A virus (A/swine/North Carolina/02023/2008(H1N1))
Influenza A virus (A/swine/Nebraska/02013/2008(H1N1))
Influenza A virus (A/swine/MN/23506/2009(H1N1))
Influenza A virus(A/swine/Iowa/46519-2/2008(H1N1))
Influenza A virus (A/swine/Ohio/02026/2008(H1N1))
Influenza A virus(A/swine/Missouri/46519-5/2009(H1N1))

Influenza A virus(A/swine/Iowa/46519-4/2008(H1N1))
Influenza A virus (A/swine/North Carolina/02084/2008(H1N1))
Influenza A virus (A/swine/Minnesota/02053/2008(H1N1))
Influenza A virus (A/swine/Iowa/02096/2008(H1N1))
Influenza A virus (A/swine/Oklahoma/011289-9/2008(H1N2))
Influenza A virus (A/swine/Oklahoma/010710-9/2008(H1N2))
Influenza A virus (A/swine/Minnesota/02011/2008(H1N2))
Influenza A virus (A/swine/Texas/01976/2008(H1N2))
Influenza A virus (A/swine/Texas/008648/2008(H1N2))
Influenza A virus (A/swine/North Carolina/R08-001877-D08-013371/2008 (H3N2))
Influenza A virus (A/swine/Kansas/015252/2009(H3N2))
Influenza A virus (A/swine/Oklahoma/042169/2008(H1N2))
Influenza A virus (A/swine/Oklahoma/011289-10/2008(H1N2))
Influenza A virus (A/swine/Oklahoma/016179-8/2008(H1N2))
Influenza A virus (A/swine/Oklahoma/016179-9/2008(H1N2))
Influenza A virus (A/swine/Oklahoma/011521-4/2008(H1N2))
Influenza A virus (A/swine/Oklahoma/011521-5/2008(H1N2))
Influenza A virus (A/swine/Oklahoma/010226-16/2008(H1N2))
Influenza A virus (A/swine/Oklahoma/010226-17/2008(H1N2))
Influenza A virus (A/swine/Oklahoma/010710-8/2008(H1N2))
Influenza A virus (A/swine/Oklahoma/020736-1/2008(H1N2))
Influenza A virus (A/swine/Oklahoma/020734-3/2008(H1N2))
Influenza A virus (A/swine/Oklahoma/011289-8/2008(H1N2))
Influenza A virus (A/swine/Texas/050593/2008(H1N2))
Influenza A virus (A/swine/Texas/050625/2008(H1N2))
Influenza A virus (A/swine/SD/31813/2009(H1N2))
Influenza A virus (A/swine/Oklahoma/032726/2008(H1N2))
Influenza A virus (A/swine/Oklahoma/053259/2008(H1N2))
Influenza A virus (A/swine/Oklahoma/020734-2/2008(H1N2))
Influenza A virus (A/swine/Oklahoma/001142/2009(H3N2))
Influenza A virus (A/swine/Oklahoma/020736-2/2008(H1N2))
Influenza A virus (A/swine/Iowa/02039/2008(H1N2))
**M (N=73)**
Influenza A virus (A/swine/IL/10-001550/2009(H1N1))
**Influenza A virus (A/swine/IL/10-001551-1/2009(H1N1))**
**Influenza A virus (A/swine/IL/10-001551-2/2009(H1N1))**
Influenza A virus (A/swine/IL/12660/2010(H1N1))
**Influenza A virus (A/swine/IL/17315-1/2010(H1N1))**
**Influenza A virus (A/swine/IL/17315-3/2010(H1N1))**
Influenza A virus (A/swine/IL/25399-2/2010(H1N1))
Influenza A virus (A/swine/IL/25399-3/2010(H1N1))
Influenza A virus (A/swine/IL/25399-4/2010(H1N1))
Influenza A virus (A/swine/IL/27486-1/2010(H1N1))
Influenza A virus (A/swine/IL/27486-2/2010(H1N1))
Influenza A virus (A/swine/IL/32974/2009(H1N1))
Influenza A virus (A/swine/IL/35572/2009(H1N1))
Influenza A virus (A/swine/IL/35573/2009(H1N1))
Influenza A virus (A/swine/IL/3910/2010(H1N1))
Influenza A virus (A/swine/IL/5265-1/2010(H1N1))
Influenza A virus (A/swine/IL/5265-2/2010(H1N1))
Influenza A virus (A/swine/IN/17311/2010(H1N1))
Influenza A virus (A/swine/Indiana/27007/2009(H1N1))
Influenza A virus (A/swine/MN/8761/2010(H1N1))
Influenza A virus (A/swine/MN/8762-1/2010(H1N1))
Influenza A virus (A/swine/MN/8762-2/2010(H1N1))
**Influenza A virus (A/swine/MO/15534/2010(H1N1))**
Influenza A virus (A/swine/MO/17314/2010(H1N1))

Influenza A virus (A/swine/MO/23881/2010(H1N1))
**Influenza A virus (A/swine/NC/13598/2010(H1N1))**
Influenza A virus (A/swine/NC/19646/2010(H1N1))
Influenza A virus (A/swine/NC/34543/2009(H1N1))
Influenza A virus (A/swine/NC/34752/2009(H1N1))
Influenza A virus (A/swine/Nebraska/02013/2008(H1N1))
Influenza A virus (A/swine/North Carolina/02023/2008(H1N1))
Influenza A virus (A/swine/North Carolina/02084/2008(H1N1))
Influenza A virus (A/swine/North Carolina/R08-001877-D08-013371/2008 (H3N2))
Influenza A virus (A/swine/Ohio/02026/2008(H1N1))
Influenza A virus (A/swine/Oklahoma/001142/2009(H3N2))
Influenza A virus (A/swine/Oklahoma/010226-16/2008(H1N2))
Influenza A virus (A/swine/Oklahoma/010226-17/2008(H1N2))
Influenza A virus (A/swine/Iowa/02039/2008(H1N2))
Influenza A virus (A/swine/Iowa/02096/2008(H1N1))
Influenza A virus (A/swine/Kansas/015252/2009(H3N2))
Influenza A virus (A/swine/Kentucky/02086/2008(H1N1))
Influenza A virus (A/swine/Minnesota/02011/2008(H1N2))
Influenza A virus (A/swine/Minnesota/02053/2008(H1N1))
Influenza A virus (A/swine/Minnesota/02093/2008(H1N1))
Influenza A virus (A/swine/Missouri/02060/2008(H1N1))
Influenza A virus (A/swine/MN/23506/2009(H1N1))
Influenza A virus (A/swine/Oklahoma/010710-8/2008(H1N2))
Influenza A virus (A/swine/Oklahoma/010710-9/2008(H1N2))
Influenza A virus (A/swine/Oklahoma/011289-10/2008(H1N2))
Influenza A virus (A/swine/Oklahoma/011289-8/2008(H1N2))
Influenza A virus (A/swine/Oklahoma/011289-9/2008(H1N2))
Influenza A virus (A/swine/Oklahoma/011521-4/2008(H1N2))
Influenza A virus (A/swine/Oklahoma/011521-5/2008(H1N2))
Influenza A virus (A/swine/Oklahoma/016179-8/2008(H1N2))
Influenza A virus (A/swine/Oklahoma/016179-9/2008(H1N2))
Influenza A virus (A/swine/Oklahoma/020734-2/2008(H1N2))
Influenza A virus (A/swine/Oklahoma/020734-3/2008(H1N2))
Influenza A virus (A/swine/Oklahoma/020736-1/2008(H1N2))
Influenza A virus (A/swine/Oklahoma/020736-2/2008(H1N2))
Influenza A virus (A/swine/Oklahoma/032726/2008(H1N2))
Influenza A virus (A/swine/Oklahoma/042169/2008(H1N2))
Influenza A virus (A/swine/Oklahoma/053259/2008(H1N2))
Influenza A virus (A/swine/OR/10-004060/2009(H1N1))
Influenza A virus (A/swine/SD/1/2010(H1N1))
Influenza A virus (A/swine/SD/31813/2009(H1N2))
Influenza A virus (A/swine/Texas/008648/2008(H1N2))
Influenza A virus (A/swine/Texas/01976/2008(H1N2))
Influenza A virus (A/swine/Texas/050593/2008(H1N2))
Influenza A virus (A/swine/Texas/050625/2008(H1N2))
Influenza A virus(A/swine/Iowa/46519-2/2008(H1N1))
Influenza A virus(A/swine/Iowa/46519-3/2008(H1N1))
Influenza A virus(A/swine/Iowa/46519-4/2008(H1N1))
Influenza A virus(A/swine/Missouri/46519-5/2009(H1N1))
**NS (N=55)**
Influenza A virus (A/swine/Arkansas/25294.25/2008(H3N2))
Influenza A virus (A/swine/Illinois/02238/2008(H1N1))
Influenza A virus (A/swine/Illinois/02251/2009(H1N1))
Influenza A virus (A/swine/Illinois/225-1/2008(H1N1))
Influenza A virus (A/swine/Iowa/02039/2008(H1N2))
Influenza A virus (A/swine/Iowa/02096/2008(H1N1))
Influenza A virus (A/swine/Iowa/225-8/2008(H1N1))

Influenza A virus (A/swine/Kansas/015252/2009(H3N2))
Influenza A virus (A/swine/Kentucky/02086/2008(H1N1))
Influenza A virus (A/swine/Minnesota/02011/2008(H1N2))
Influenza A virus (A/swine/Minnesota/02053/2008(H1N1))
Influenza A virus (A/swine/Minnesota/02093/2008(H1N1))
Influenza A virus (A/swine/Minnesota/225-11/2008(H1N1))
Influenza A virus (A/swine/Minnesota/225-3/2008 (H1N1))
Influenza A virus (A/swine/Minnesota/225-4/2008(H1N1))
Influenza A virus (A/swine/Minnesota/225-5/2008 (H1N1))
Influenza A virus (A/swine/Minnesota/61598.3/2008(H1N1))
Influenza A virus (A/swine/Missouri/02060/2008(H1N1))
Influenza A virus (A/swine/MN/23506/2009(H1N1))
Influenza A virus (A/swine/Nebraska/02013/2008(H1N1))
Influenza A virus (A/swine/North Carolina/02023/2008(H1N1))
Influenza A virus (A/swine/North Carolina/02084/2008(H1N1))
Influenza A virus (A/swine/North Carolina/225-12/2008(H1N1))
Influenza A virus (A/swine/North Carolina/225-13/2008(H1N1))
Influenza A virus (A/swine/North Carolina/225-2/2008(H1N1))
Influenza A virus (A/swine/North Carolina/225-6/2008(H1N1))
Influenza A virus (A/swine/North Carolina/225-7/2008(H3N2))
Influenza A virus (A/swine/North Carolina/R08-001877-D08-013371/2008 (H3N2))
Influenza A virus (A/swine/Ohio/02026/2008(H1N1))
Influenza A virus (A/swine/Ohio/02090/2008(H1N1))
Influenza A virus (A/swine/Oklahoma/001142/2009(H3N2))
Influenza A virus (A/swine/Oklahoma/010226-16/2008(H1N2))
Influenza A virus (A/swine/Oklahoma/010226-17/2008(H1N2))
Influenza A virus (A/swine/Oklahoma/010710-8/2008(H1N2))
Influenza A virus (A/swine/Oklahoma/010710-9/2008(H1N2))
Influenza A virus (A/swine/Oklahoma/011289-10/2008(H1N2))
Influenza A virus (A/swine/Oklahoma/011289-8/2008(H1N2))
Influenza A virus (A/swine/Oklahoma/011289-9/2008(H1N2))
Influenza A virus (A/swine/Oklahoma/011521-4/2008(H1N2))
Influenza A virus (A/swine/Oklahoma/011521-5/2008(H1N2))
Influenza A virus (A/swine/Oklahoma/016179-8/2008(H1N2))
Influenza A virus (A/swine/Oklahoma/016179-9/2008(H1N2))
Influenza A virus (A/swine/Oklahoma/020734-2/2008(H1N2))
Influenza A virus (A/swine/Oklahoma/020734-3/2008(H1N2))
Influenza A virus (A/swine/Oklahoma/020736-1/2008(H1N2))
Influenza A virus (A/swine/Oklahoma/020736-2/2008(H1N2))
Influenza A virus (A/swine/Oklahoma/02530/2009(H3N2))
Influenza A virus (A/swine/Oklahoma/032726/2008(H1N2))
Influenza A virus (A/swine/Oklahoma/042169/2008(H1N2))
Influenza A virus (A/swine/Oklahoma/053259/2008(H1N2))
Influenza A virus (A/swine/Oklahoma/225-14/2008(H3N2))
Influenza A virus (A/swine/Texas/008648/2008(H1N2))
Influenza A virus (A/swine/Texas/01976/2008(H1N2))
Influenza A virus (A/swine/Texas/050593/2008(H1N2))
Influenza A virus (A/swine/Texas/050625/2008(H1N2))

*www.ncbi.nlm.nih.gov/genomes/FLU/FLU.html; Date: 28th Oct 2010. Based on the primer and probe sequences, no false positive samples is expected to be observed, and viral sequences predicted to have false negative results (N=23) are in **boldface**.

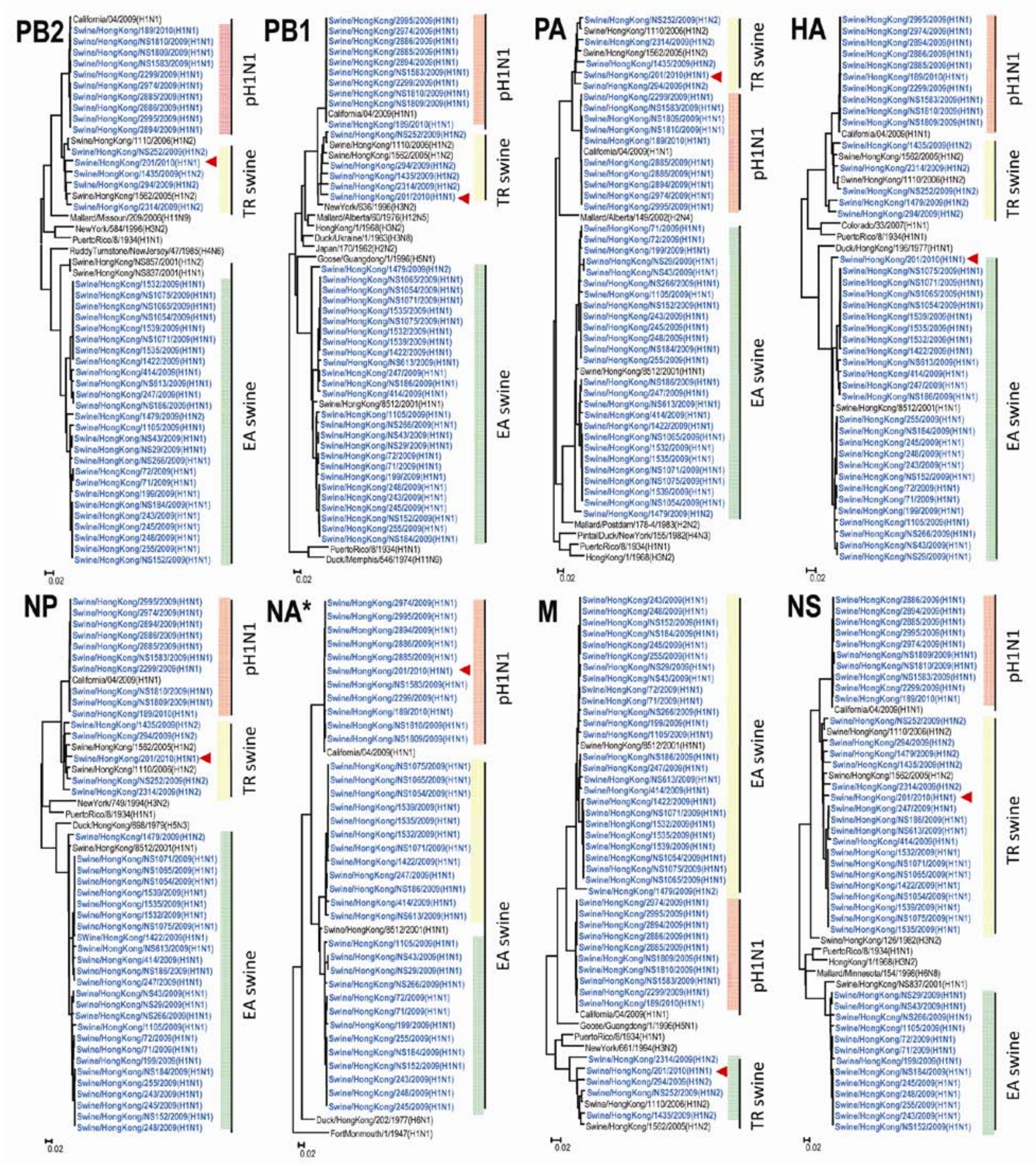

Technical Appendix Figure 1. Genotyping of swine influenza viruses. Swine influenza viruses tested by the genotyping assays. Swine influenza viruses tested in this study are highlighted in blue. Representative human, avian, classical swine, Eurasian avian-like (EA) swine and triple reassortant (TR) swine influenza virus sequences from GenBank (black) are included as references. Sequences from viruses determined in this study were described elsewhere (*5,6*). Viruses yielding double positive, double negative and SYBR green–positive/cyanine 5–negative signals are highlighted in red, green, and yellow,

respectively. The reassortant of pandemic (H1N1) (A/swine/Hong Kong/201/2010) is highlighted by an arrow in each tree. *N2 sequences were excluded from the analysis. Phylogenetic trees of swine influenza viruses were constructed by the neighbor-joining method. Scale bar indicates estimated genetic distance of these viruses.

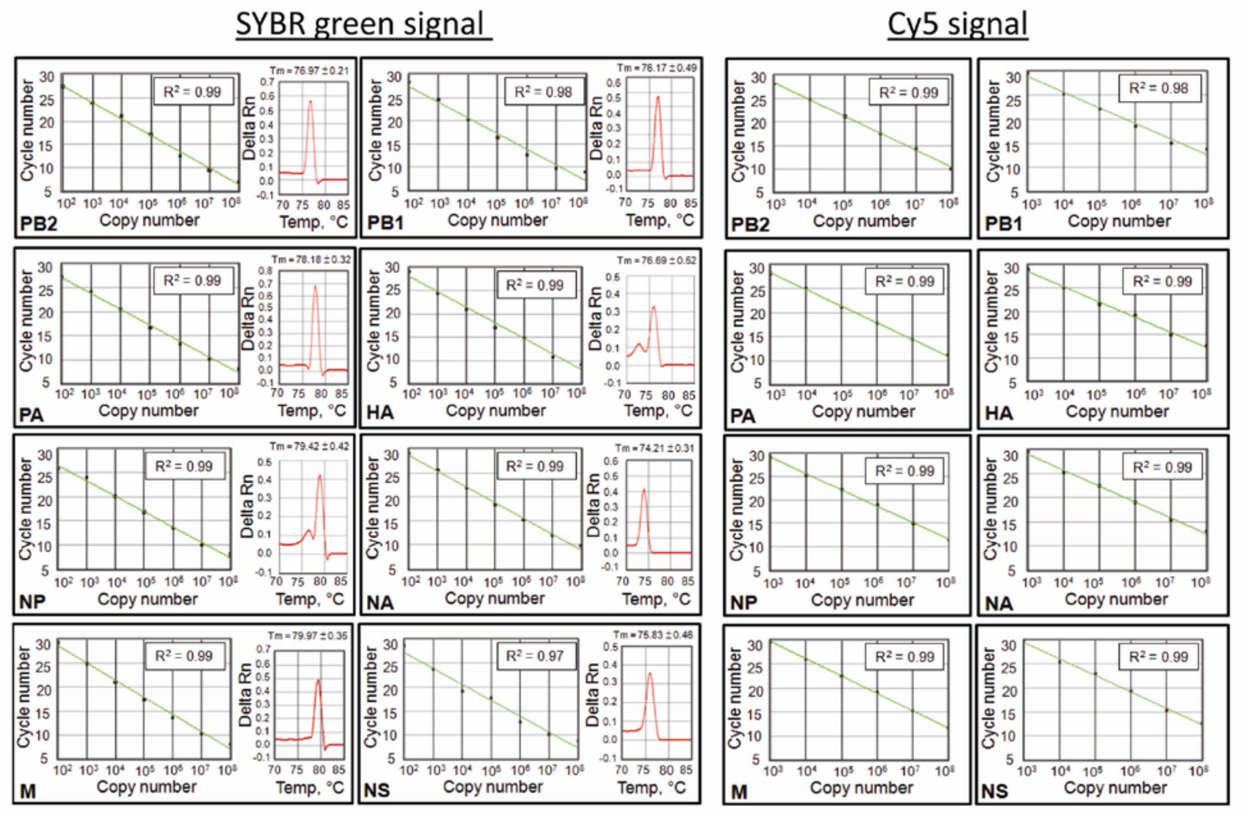

Technical Appendix Figure 2. Combined SYBR green/hydrolysis probe quantitative RT-PCR assays for genotyping of swine influenza virus. Dynamic ranges of the segment-specific assays deduced from the SYBR green signal (left) and Cy5 (right) signals are shown. The square of correlation coefficient (R^2) of the standard curves are indicated. The melting curves observed from the SYBR signals are shown (left) and the melting temperature (Tm, average ± SD) of pandemic H1N1 gene segments were deduced from ≥20 independent reactions.
